# Gut microbiota mediates semaglutide attenuation of diabetes-associated cognitive decline

**DOI:** 10.1016/j.neurot.2025.e00615

**Published:** 2025-05-23

**Authors:** Liqin Qi, Huimin Kang, Feihui Zeng, Menglan Zhan, Cuihua Huang, Qintao Huang, Lijing Lin, Guanlian He, Xiaoying Liu, Xiaohong Liu, Libin Liu

**Affiliations:** a Department of Endocrinology, Fujian Institute of Endocrinology, Fujian Medical University Union Hospital, Fuzhou, Fujian, People's Republic of China; b Department of Pediatrics, Fujian Medical University Union Hospital, Fuzhou, Fujian, People's Republic of China

**Keywords:** Semaglutide, Cognitive impairment, Gut microbiota, Neuroactive ligand-receptor interactions, Metabolome

## Abstract

Diabetes-associated cognitive decline (DACD), characterized by cognitive impairment, is a serious complication of diabetes mellitus (DM). Research has shown that semaglutide, a novel glucagon-like peptide-1 receptor agonist, has neurotrophic and neuroprotective properties. However, a comprehensive understanding of the specific effects and underlying mechanisms of semaglutide treatment in patients with DACD remains lacking. In this study, we evaluated the potential of semaglutide to alleviate DACD in mice with DM. Eight-week-old mice fed a high-fat diet with streptozotocin-induced DM were subcutaneously injected with semaglutide (30 ​nmol/kg qd) for 12 weeks. Semaglutide administration significantly alleviated cognitive impairment, inhibited hippocampal neuron loss, improved the hippocampal synaptic ultrastructure, and effectively mitigated neuroinflammation. Furthermore, semaglutide treatment increased the relative abundances of *g_Alistipes, g_norank_f_Eubacterium_coprostanoligenes, g_Bacteroides,* and *g_Parabacteroides*, while decreasing the relative abundances of *g_ faecalibaculum, g_Colodertribacter, g_GCA-900066575, g_Erysipelatoclostridium,* and *g_norank_f_Lachnospiraceae*. Semaglutide also induced alterations in fecal and serum metabolites, as well as transcriptomic changes in brain tissue, with significant common enrichment in neuroactive ligand-receptor interactions. Furthermore, strong correlations were observed among semaglutide-affected genes, metabolites, and microbiota, as assessed by correlation analysis and integrative modeling. In conclusion, these findings suggest a correlation between the protective effects of semaglutide against DACD and the microbiota-gut-brain axis.

## Graphical abstract

**Figure:**
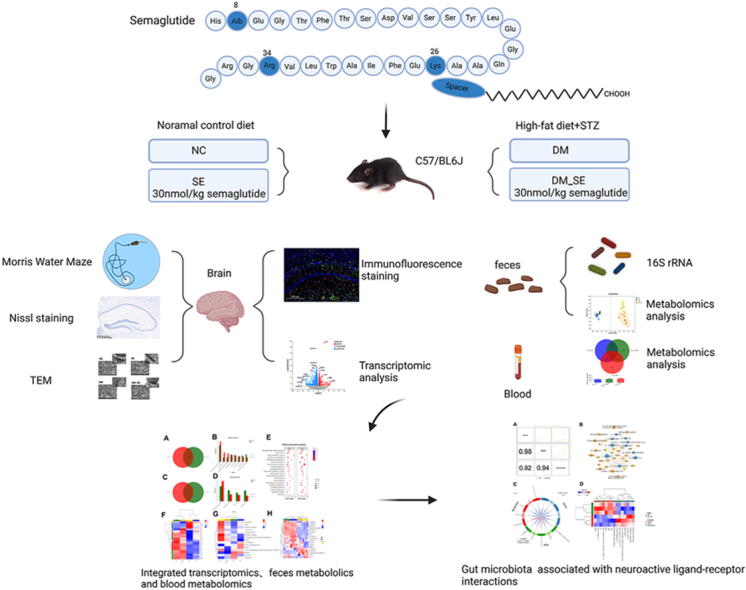

## Introduction

The global population affected by diabetes mellitus (DM) is estimated to be approximately 500 million, leading to significant challenges for society, the economy, and families [1]. Diabetes-associated cognitive decline (DACD) is a serious complication of DM characterized by cognitive impairment, memory deficits, and neuronal loss [2]. The mechanisms underlying DACD include brain insulin resistance, hyperglycemia, hypoglycemia, oxidative stress, cerebral microvascular dysfunction, neuroinflammation, and microbiota dysbiosis [3]. Although understanding these potential mechanisms can aid in developing treatment strategies for DACD, there is still no effective strategy to halt its progression.

The “gut-brain” axis is a multiple biological communication system that facilitates bidirectional interactions between the gut microbiota and the brain [4]. It plays a pivotal role in maintaining both the gut microenvironment and central nervous system, making it a key area of research related to neurological disorders. The three primary methods of bidirectional communication include neuronal pathways, impact on metabolism, and immune system modulation [5]. Increasing evidence suggests that the gut microbiota may mediate the development and progression of neurodegenerative disorders, including Parkinson's disease (PD) [6], Alzheimer's disease (AD) [7], and DACD [8,9]. Clinical studies have shown that patients with diabetes often exhibit intestinal mucosal injury and microbial dysbiosis [[10], [11], [12]]. These findings indicate that correcting the intestinal microenvironment disorders caused by DM may be crucial for treating DACD. Therefore, targeting the “microbiota-gut-brain axis (MGBA)” has become an important research focus for developing potential therapeutic strategies for DACD.

Semaglutide, a glucagon-like peptide-1 receptor agonist (GLP-1RA), treats type 2 DM [13]. Emerging evidence suggests that semaglutide exerts neuroprotective effects [14]. Semaglutide has been shown to reduce cognitive impairment induced by a high-fat diet (HFD) in obese mice by increasing the phosphorylation of proteins related to neuronal development and synaptic plasticity [15]. In addition, semaglutide enhanced the cognitive abilities of rats with type 2 diabetes-related depression, protected synaptic plasticity, and reduced neuroinflammation [16]. Our previous study revealed that liraglutide, another GLP-1RA, increased the support provided by astrocytes to neurons via promoting aerobic glycolysis and decreased p-tau levels by inhibiting glycogen synthase kinase-3β (GSK-3β) [17,18]. Moreover, regarding the gut microbiota, semaglutide has been shown to alleviate gut microbiota dysbiosis associated with non-alcoholic fatty liver disease and restore the abundance of *Akkermansia, Faecalibaculum, Allobaculum, Lachnospiraceae* and *Bacteroides* in obese mice [19,20]. However, the mechanisms through which semaglutide modulates MGBA and improves DACD pathology remain unclear.

In this study, we investigated the effects and underlying mechanisms of semaglutide treatment on cognitive function in HFD-fed, streptozotocin (STZ)-induced DM mice. We evaluated pathological changes and neuroinflammation in the brain following semaglutide treatment. The mechanisms through which semaglutide regulates DACD were explored using a combination of the microbiome, fecal metabolome, blood metabolome, and brain transcriptomic analyses. This study provides new insights for the prevention and treatment of DACD.

## Material and Methods

### Animals

Eight-week-old male C57BL/6 mice were purchased from Beijing Huafukang Biotechnology Co., Ltd. [Animal certificate number: SCXK (Jing) 2019-0008]. All animals were housed in a pathogen-free facility (ICV system, Tecniplast, Italy) and maintained in a stable environment with a temperature of 23 ​± ​1 ​°C, 50–60 ​% humidity, and a 12 ​h light/dark cycle with free access to food and clean tap water.

The animals underwent a 1-week adaptive feeding period before the experiment. Mice were then randomly divided into four groups: normal control (NC), normal control with semaglutide (SE), diabetes mellitus (DM), and diabetes mellitus with semaglutide (DM_SE). Each group consisted of 12 mice. Mice in the DM and DM_SE groups were fed HFD (60 ​% fat) (TP23400, TROPHIC Animal Feed High-Tech Co. LTD, China) in the 2nd −27th weeks to induced DM, while those in the NC and SE groups were fed a normal control diet. In the 14th-15th weeks, mice in the DM and DM_SE groups received intraperitoneal injections of STZ at 40 ​mg/kg for 5 consecutive days, while mice in the NC and SE groups received an equal dose of physiological saline. Blood samples were collected from the tail tip for random blood glucose testing on days 3, 5, and 7 after the final injection of STZ.A blood glucose level ≥16.7 ​mmol/L on at least two occasions confirmed successful induction of the diabetes model. Afterward, in the 16th-27th weeks, all animals in the SE and DM_SE groups received subcutaneous injections of semaglutide (30 ​nmol/kg, qd) for 12 weeks, while animals in the NC and DM groups received vehicle injections. The semaglutide dose was gradually increased over the first 6 days (0.6, 1.2, 2.4, 4.8, 12, 30 ​nmol/kg) to mitigate temporary discomfort induced by GLP-1RA treatment [21]. The final dose was maintained for the remainder of the treatment [22].

**Fig. 1:**
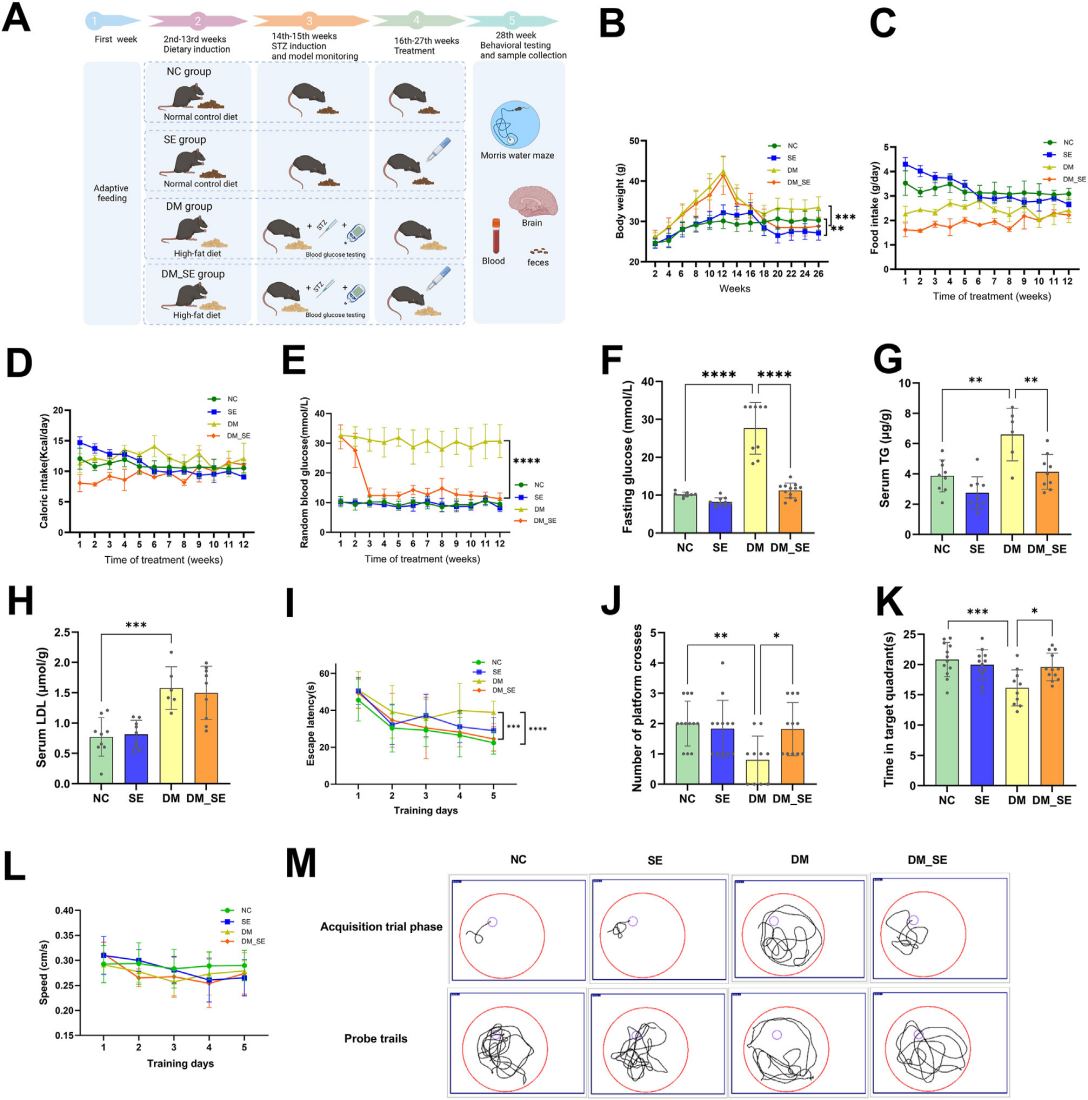
Semaglutide treatment improved learning and memory impairments in DM mice. (A) Timeline of the study and experimental design. (B) Body weight of each group (n ​= ​10–12 mice per group). (C) Food intake. (D) Caloric intake. (E) Random blood glucose levels across weeks. (F) Fasting blood glucose levels. (G) Serum TG concentration (H) Serum LDL-C concentration (n ​= ​6–8 mice per group). (I) Escape latency during the 5-day training trial in the Morris water maze test. (J) Number of platform crossings. (K) Time spent in the target quadrant. (L) Swimming speed in the spatial exploration test. (M) Representative swimming tracks of mice during the acquisition trial phase on day 5 and probe trials (n ​= ​10–12 mice per group). Abbreviations: NC: normal control group; SE: normal control with semaglutide group; DM: Diabetes mellitus group; DM_SE: Diabetes mellitus with semaglutide group. Data are presented as mean ​± ​SD. ∗*p* ​< ​0.05, ∗∗*p* ​< ​0.01, ∗∗∗*p* ​< ​0.001, ∗∗∗∗*p* ​< ​0.0001.

Body weight was monitored weekly throughout the experiment. Random blood glucose levels from the tail vein and food intake were measured weekly following the initiation of semaglutide treatment. Food intake was calculated as the mean daily consumption per mouse. The experimental groups and treatment protocols are illustrated in Fig. 1A. All animal procedures were complied with ARRIVE guidelines and approved by the Institutional Animal Care and Use Committee of Fujian Medical University (IACUC FJMU). [Approval Number: IACUC FJMU 2022-0807].

### Morris water maze (MWM)

The learning and memory abilities of the mice were assessed using the MWM test, as described in our previous study [18]. Briefly, all mice underwent four trials per day with at least 10-min intervals for 5 consecutive days during the acquisition phase. Mice were allowed 60 ​s to find the submerged platform. If a mouse failed to locate the platform within 60 ​s, it was placed on the platform for 10 ​s. The escape latency was defined as the time required to find the platform within 60 ​s. Escape latency and average swimming speed were recorded for each trial. On day 6, a “probe trial” was conducted with the submerged platform removed. The mice were allowed to swim freely for 60 ​s, and the number of crossings over the target quadrant and time spent in the target quadrant were recorded.

### Sample collection

The mice were anesthetized with 2 ​% pentobarbital sodium (50 ​mg/kg, intraperitoneally). Blood samples were collected by enucleating the eyeball, centrifuged at 3000 r/min for 10 ​min at 4 ​°C, and stored at −80 ​°C. The skulls were then removed, and the brains were rapidly placed on ice. Four brains from each group were split in half; the left hemispheres were fixed in 4 ​% paraformaldehyde, and the right hippocampus was dissected and fixed using fixative (G1102, Servicebio, China) for transmission electron microscopy (TEM). The remaining cerebral cortex and hippocampus were dissected on ice. The brain samples were snap-frozen in liquid nitrogen and stored at −80 ​°C for further analysis, including metabolomic analysis and enzyme-linked immunosorbent assay (ELISA).

### Biochemical analyses

Fasting glucose, serum triglyceride (TG), and low-density lipoprotein cholesterol (LDL-C) levels were detected using commercial kits (RXWB0458-96 Ruixinbio, Quanzhou, China), TG Content assay kits (RXWB0011-96 Ruixinbio, Quanzhou, China), and LDL-C Content assay kits (RXWB0792-96 Ruixinbio, Quanzhou, China), respectively, according to the manufacturer's instructions.

### Histology and immunofluorescence

Paraffin-embedded tissues were sectioned into 5 ​μm-thick slices. After dewaxing and dehydration, the sections were stained with a Nissl staining solution. Subsequently, cells were differentiated using 95 ​% alcohol, dehydrated with anhydrous alcohol, permeabilized with xylene, and sealed with a neutral resin. Images of the Nissl staining were observed under a microscope (Eclipse 50i, Nikon, Japan).

Double immunofluorescence staining was performed on the brain slices. Non-specific binding was blocked using 5 ​% bovine serum albumin (BSA, B2064, Sigma) for 20 ​min at room temperature (RT) before incubation at 4 ​°C overnight with primary antibodies (anti-GFAP, 1:400, ab68428, Abcam, USA; anti-Iba-1, 1:200, ab178847, USA). After washing with phosphate-buffered saline (PBS), the slices were incubated with a secondary antibody (1:200, ab150077, Abcam) for 30 ​min at 37 ​°C. The slices were then incubated with try-594 tyramine conversion reagent for 20 ​min at RT. Subsequently, for double staining, antigen retrieval and incubation were repeated, and the sections were further incubated with try-488 tyramine conversion reagent for 20 ​min at RT. Nuclei were counterstained with 4′,6-diamidino-2-phenylindole (DAPI) (P0131, Beyotime Biotechnology, Jiangsu, China). Immunofluorescent signal images were acquired using a digital slide scanner (3DHISTECH; PANNORAMIC SCAN II, Budapest, Hungary). Fluorescence signals were observed by ImageJ software (NIH, Bethesda, MD, USA).

### Transmission electron microscope (TEM)

The CA1 region of the right hippocampus was cut into 1 ​mm^3^ cubes, which were fixed by immersion in 2.5 ​% buffered glutaraldehyde at 4 ​°C. After washing with 0.1 ​M PBS (pH 7.4), the specimen was postfixed with 1 ​% osmic acid at RT 2 ​h in the dark, and then in a 1 ​% osmium tetroxide solution. Grades of ethanol and acetone were used for dehydration. Subsequently, the specimen was resin-penetrated and embedded using acetone and EMBed 812. The resin blocks were cut into 70 ​nm thick sections using an ultramicrotome (Leica UC7, Leica, Germany). The slices were stained with 2 ​% uranyl acetate and 2.6 ​% lead citrate. The sections were observed using a TEM (HT7800, Hitachi, Japan).

### Gut microbiota analysis

Before sacrificing the mice, fresh fecal samples were collected, snap-frozen in liquid nitrogen, and then stored at −80 ​°C. Total genomic DNA was extracted using a PF Mag-Bind Stool DNA Kit (M9636-02) (Omega Bio-Tek, Norcross, GA, USA) according to the manufacturer's instructions. After determining the DNA concentration and integrity, the 16S rRNA gene, comprising hypervariable regions V3–V4, was amplified using a pair of primes (338F: 5′-ACTCCTACGGGAGGCAGCAG-3’; 806R: 5′-GGACTACHVGGGTWTCTAAT-3′) to enrich the target fragment for each fecal sample. The PCR products were sequenced using the Illumina PE300 platform (Illumina, San Diego, CA, USA) according to the manufacturer's instructions. FastP and FLASH software were used to filter and merge raw sequencing data. High-quality sequences were denoised using the DADA2 plugin in the Qiime2 pipeline (version 2020.2). DADA2-denoised sequences are known as amplicon sequence variants (ASVs). Species taxonomic analysis of the ASVs was performed using the naïve Bayes classifier in Qiime2.

### Metabolomics analysis

Metabolomic profiling analysis was performed using blood and fecal samples, as previously described [23]. Briefly, blood samples were mixed with a 4-fold volume of extraction solution (acetonitrile: methanol ​= ​1:1), subjected to low-temperature ultrasonic extraction, and the supernatant was analyzed after centrifugation. For fecal samples, 400 ​μL of extraction solution (methanol: water ​= ​4:1) was added to 50 ​g of fecal material, ground, extracted with low-temperature ultrasound, and centrifuged and the supernatant was collected for analysis. The LC-MS/MS analysis was performed on a Thermo UHPLC-Q Exactive HF-X system equipped with an ACQUITY HSS T3 column (100 ​mm ​× ​2.1 ​mm i.d., 1.8 ​μm; Waters, USA). The raw data generated were converted into a common format using Progenesis QI software (Waters, Milford, USA) through baseline removal, smoothing and peak values, and alignment. Metabolites were identified by searching databases, including the HMDB (http://www.hmdb.ca/) and Metlin (https://metlin.scripps.edu/). Principal component analysis (PCA) and orthogonal least partial squares discriminant analysis (OPLS-DA) were conducted using the R package “ropls” (Version 1.6.2). The Kyoto Encyclopedia of Genes and Genomes database (KEGG, http://www.genome.jp/kegg/) was used for the enrichment analysis of differential metabolites.

### Transcriptomic analysis

Total RNA was extracted from the cerebral cortex using TRIzol Reagent (T01-500, Yuhua Biomedical Technology Co., Ltd., Majorbio, Shanghai, China) following the manufacturer's protocol. RNA concentration and purity were quantified using a Nanodrop-2000 system (Thermo Scientific, Waltham, MA, USA). RNA purification, library preparation, and transcriptome sequencing were performed by Shanghai Bio-Pharm Biotechnology Co. Ltd. (Majorbio, Shanghai, China). Briefly, 1 ​μg of total RNA was used for library construction with the Illumina® Stranded mRNA Prep Ligation Kit (20040534, Illumina, San Diego, CA, USA), according to the manufacturer's protocol. After quality assessment using Qubit 4.0, paired-end reads were generated using the NovaSeq Xplus sequencer platform (Illumina, San Diego, CA, USA). Raw data were trimmed and quality controlled via fastp using default parameters [24]. Subsequently, the clean reads were separately aligned to the reference genome of mice using HISAT2 software [25]. The mapped reads of each sample were analyzed using StringTie [26]. The DESeq2 software was used to perform differential analysis of gene expression between samples [27]. Genes with p ​< ​0.05 and an absolute value of log2 fold change (|log2FC|) ​> ​1.2 were considered significantly differentially expressed genes (DEGs). GO functional enrichment and KEGG pathway analyses were performed to further analyze the DEGs.

### Statistical analysis

Data are presented as mean ​± ​standard deviation (SD). Statistical analyses were performed using GraphPad Prism 9.4.0 (GraphPad Software, California, USA). Two-way analysis of variance, followed by Tukey's post-hoc multiple comparison test, was applied for the data analysis. Statistical significance was set at *p* ​< ​0.05.

## Results

### Effect of semaglutide on STZ-induced diabetes in mice

The grouping and treatment of the mice are shown in Fig. 1A. As shown in Fig. 1B, the body weight of mice in the DM group and the DM-SE group significantly increased (16.36 ​± ​2.87 ​g, 15.35 ​± ​3.17 ​g, respectively) after induction with HFD. However, after 12 weeks of treatment with semaglutide, the weight loss in the DM-SE group (5.55 ​± ​2.97 ​g) was significantly greater than that of the DM group (2.78 ​± ​1.51 ​g) (*p* ​< ​0.001). The body weight of the SE group decreased (4.39 ​± ​2.05 ​g), while the NC group showed a slight increase in body weight (1.02 ​± ​0.73 ​g). In terms of food intake, the initial intervention with semaglutide reduced the amount of food intake in mice, but there was no significant difference in food intake between the groups after 12 weeks of intervention (Fig. 1C, *p* ​> ​0.05). Similar results were observed for caloric intake (Fig. 1D, *p* ​> ​0.05). Semaglutide significantly reduced blood glucose levels of diabetic mice (Fig. 1E, *p* ​< ​0.05) but had no significant effect on NC mice (Fig. 1E, *p* ​> ​0.05). Fig. 1F shows the fasting blood glucose levels of mice after semaglutide intervention. Blood glucose levels in the DM group were significantly higher than those in the NC group (Fig. 1F, *p* ​< ​0.05). After semaglutide treatment, blood glucose levels significantly decreased (Fig. 1F, *p* ​< ​0.05). The levels of triglycerides (TG) and low-density lipoprotein cholesterol (LDL) in the mouse serum increased in the DM group (Fig. 1G, *p* ​< ​0.01; Fig. 1H, *p* ​< ​0.001). Semaglutide intervention significantly reduced TG levels in DM mice (Fig. 1G, *p* ​< ​0.01); however, there was no significant difference in LDL levels (Fig. 1H, *p* ​> ​0.05).

### Semaglutide improves cognitive performance of DM mice

To evaluate the effects of semaglutide on memory in DM mice, we used the MWM to assess spatial learning and memory. As shown in Fig. 1I, compared to the control group, the average escape latency in the hidden platform test of HFD- and STZ-induced DM mice was significantly increased (*p* ​< ​0.0001). In addition, the average escape latency of the DM_SE group was partly preserved compared with that of DM mice (*p* ​< ​0.001). As expected, semaglutide did not affect escape latencies in the search for hidden platforms in normal mice. In the probe trial, with the platform removed, the number of platform crossings varied significantly among the four groups. Mice in the DM group had fewer platform crossings over the area where the platform was previously located (Fig. 1J, *p* ​< ​0.01) than mice in the NC group. After semaglutide treatment, the number of platform crossings increased (Fig. 1J, *p* ​< ​0.05). We calculated the time spent in the target quadrant during the probe trial and found that the DM group mice spent less time than the NC group mice (Fig. 1K, *p* ​< ​0.001), while the DM-SE group mice spent more time in the target quadrant than the DM group mice (Fig. 1K, *p* ​< ​0.05). The data indicated that semaglutide partially preserved the cognitive impairment induced by HFD and STZ. The swimming speed data among the four groups were not significantly different (Fig. 1L, *p* ​> ​0.05), indicating no significant difference in motor abilities among the groups. Typical swimming trajectories for each group of mice in the acquisition and probe trials are shown in Fig. 1M.

### Semaglutide alleviates pathological changes of the hippocampus in DM mice

**Fig. 2:**
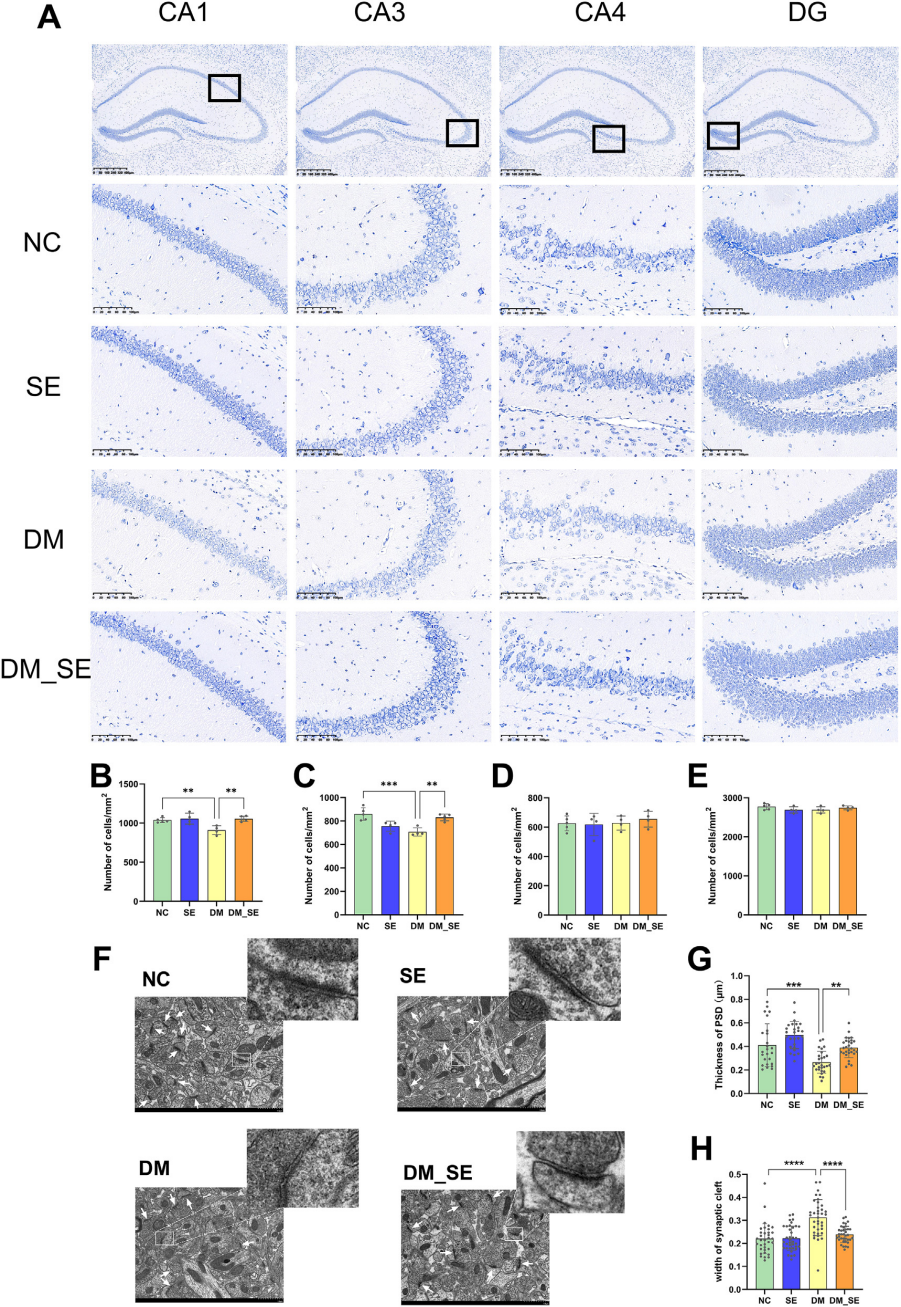
Effect of semaglutide treatment on neurons and synapsis in the hippocampus. (A) Representative graphs of Nissl staining in the hippocampus (n ​= ​4 per group). (B–E) Number of neurons in CA1 region (B), CA3 region (C), CA4 region (D), and DG region (E). (F) Ultrastructure of chemical synapses in hippocampal CA1 region (n ​= ​3 per group). (G) Thickness of postsynaptic density (PSD)(H) Width of the synaptic cleft. White arrow: synapse. Abbreviations: NC: normal control group; SE: normal control with semaglutide group; DM: Diabetes mellitus group; DM_SE: Diabetes mellitus with semaglutide group. Data presented as mean ​± ​SD. ∗*p* ​< ​0.05, ∗∗*p* ​< ​0.01, ∗∗∗*p* ​< ​0.001, ∗∗∗∗*p* ​< ​0.0001.

The number of hippocampal neurons in the CA1 and CA3 regions was significantly lower in the DM group than in the NC group (Fig. 2A–C), whereas no significant differences were observed in the CA4 and DG subregions of the DM groups (Fig. 2D–E, *p* ​> ​0.05, *p* ​> ​0.05). Semaglutide treatment significantly prevented the loss of hippocampal neurons in the CA1 and CA3 subregions in DM mice compared to that in the NC group (Fig. 2B–C, *p* ​< ​0.05, *p* ​< ​0.05). However, the number of hippocampal neurons in the CA4 and DG subregions did not increase after semaglutide treatment in DM mice (Fig. 2D–E, *p* ​> ​0.05, *p* ​> ​0.05).

Transmission electron microscopy was used to observe the ultrastructure of synapses in the CA1 region of each group of mice. Fig. 2F shows typical synaptic structures in each group. The white arrows indicate the synapses. In the NC and SE groups, the presynaptic and postsynaptic membranes were intact and consecutive. The postsynaptic membrane had many dense (PSD) substances, and synaptic vesicles contained abundant neurotransmitters. The synaptic cleft was narrow and parallel to the postsynaptic membrane. However, in the DM group, as shown in Fig. 2F, the synaptic structure was altered, with a partial fracture of the presynaptic and postsynaptic membranes, significant reduction in PSD, and widening of the synaptic cleft. These changes in the chemical synapses of DM mice suggest decreased cognitive memory. In the DM_SE group, the synaptic structure was partially restored, with evenly distributed vesicles, more regular presynaptic and postsynaptic membranes, slightly thickened PSD, and a narrower synaptic cleft compared to the DM group. Fig. 2G and H show the statistical analysis of PSD thickness and synaptic cleft width, respectively. As expected, the PSD thickness of the DM mice decreased (*p* ​< ​0.001), and the synaptic cleft width increased (*p* ​< ​0.0001). Semaglutide treatment partially reversed these trends (*p* ​< ​0.01 and *p* ​< ​0.0001, respectively). These results suggest that semaglutide protects the structure of chemical synapses in the CA1 region of mice.

**Fig. 3:**
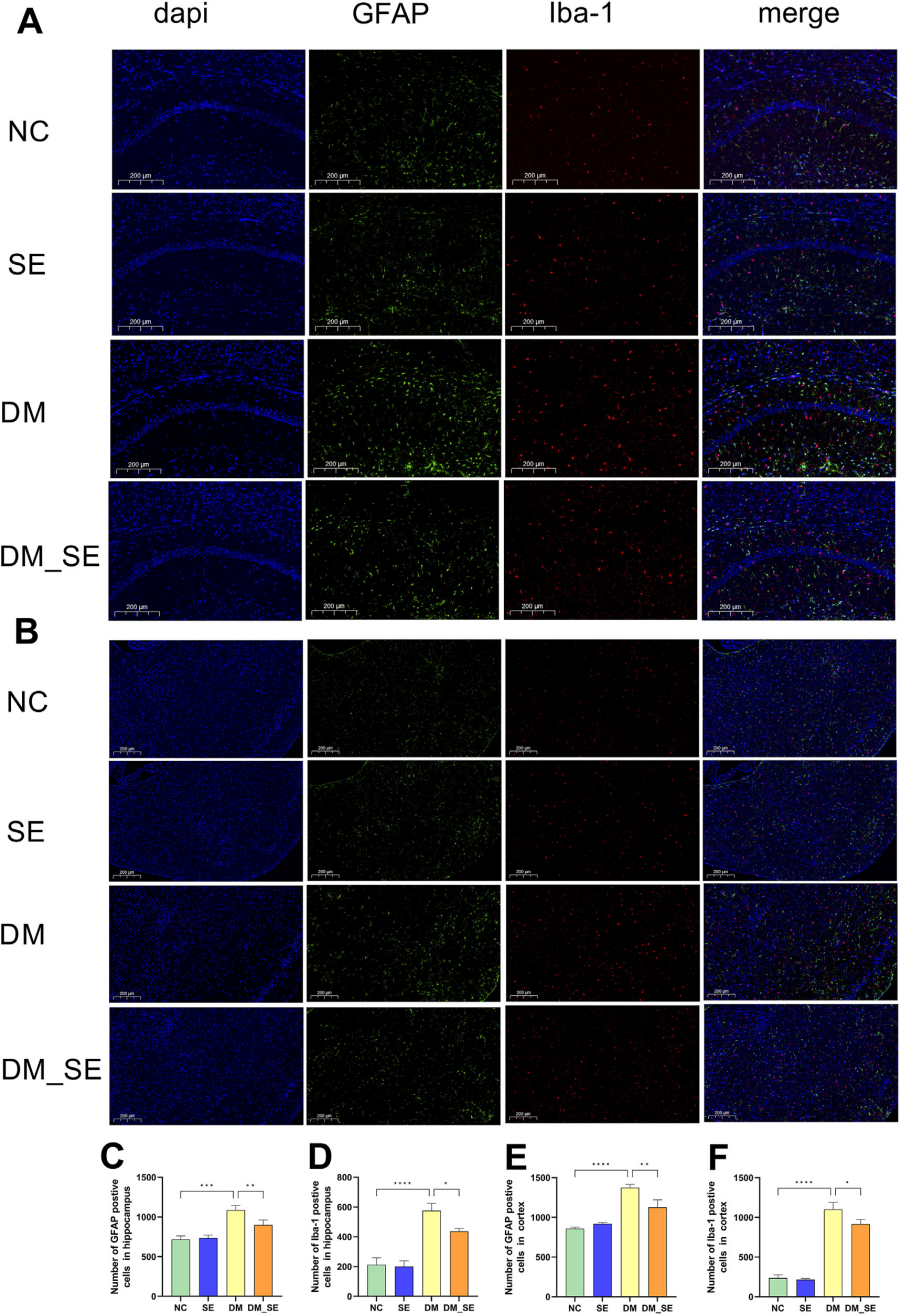
Effect of semaglutide treatment on neuroinflammation. (A) Immunofluorescent images of GFAP (green)/Iba-1 (red) colocalization in the hippocampus. (B) Immunofluorescent images of GFAP (green)/Iba-1 (red) colocalization in the cortex. (n ​= ​3/group). (C–D) Number of GFAP/Iba-1 positive cells in the hippocampus. (E–F) Number of GFAP/Iba-1 positive cells in the cortex. (n ​= ​3/group). Abbreviations: NC: normal control group; SE: normal control with semaglutide group; DM: Diabetes mellitus group; DM_SE: Diabetes mellitus with semaglutide group. Data presented as mean ​± ​SD. ∗*p* ​< ​0.05, ∗∗*p* ​< ​0.01, ∗∗∗*p* ​< ​0.001, ∗∗∗∗*p* ​< ​0.0001.

In addition, immunofluorescence of GFAP and Iba-1 was used to investigate the activation of astrocytes and microglia. As shown in Fig. 3A and B, heightened GFAP and Iba-1 were observed in the hippocampus and cortex of the DM group compared to the NC and SE groups. However, the increase in the number of GFAP and Iba-1 positive cells was substantially reduced in the semaglutide-treated mice in both the hippocampus and cortex (Fig. 3C–F). These results suggest that semaglutide effectively attenuates neuroinflammation in DM mice.

### The effect of semaglutide treatment on gut microbiota composition

**Fig. 4:**
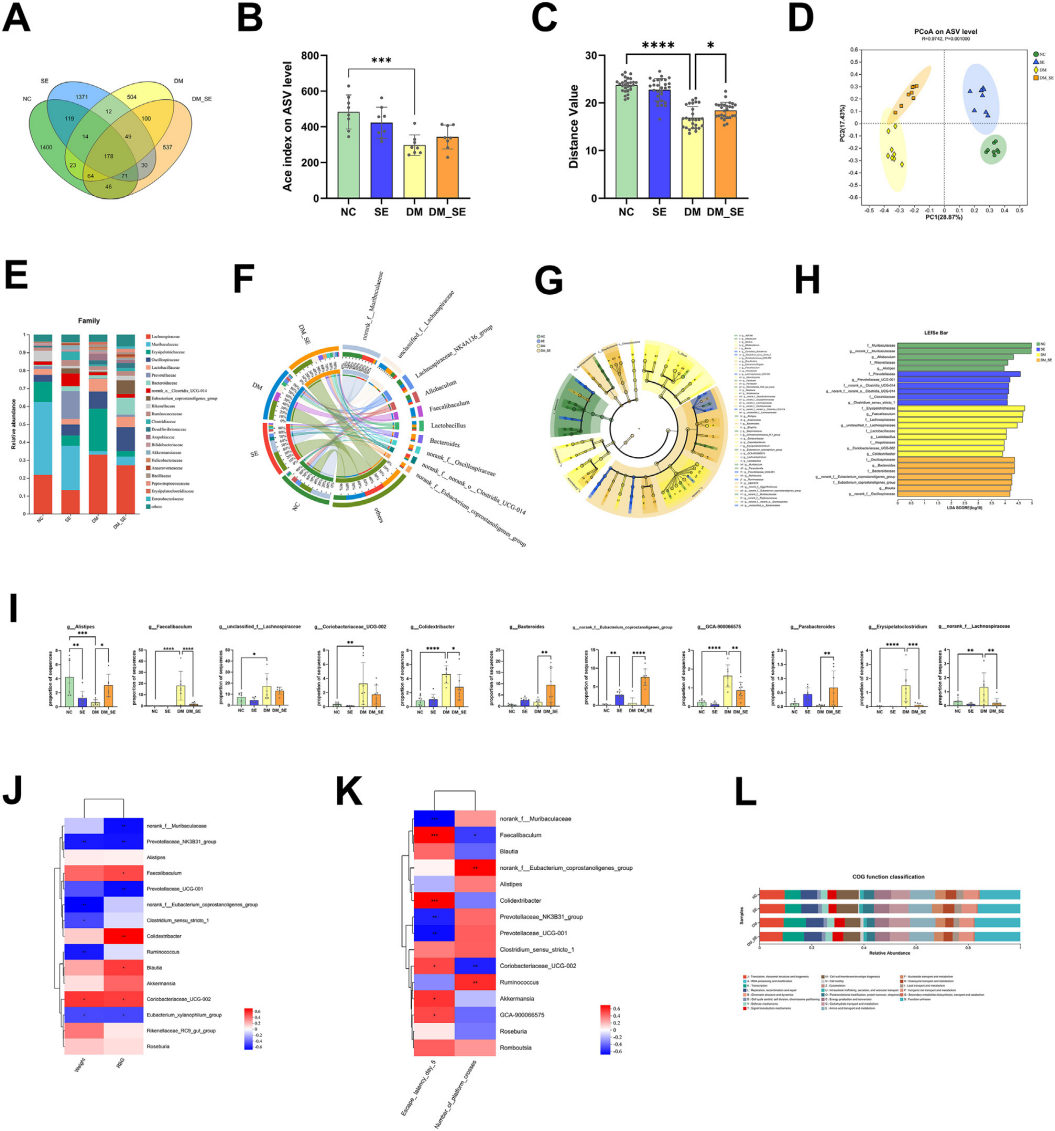
Semaglutide treatment modified the composition of gut microbiota in DM mice. (A) Venn diagrams of unique ASV in different groups. (B) α-diversity analysis. (C) β-diversity differences analysis. (D) PCoA analysis at the ASV level. (E) Relative abundance (%) at the family level in each group. (F) Main microbial composition of each group at the family level. (G) Clustering tree of LEfSe comparison from the domain to the genus level. (H) LDA analysis. (I) Relative abundance of representative gut microbiota at genus level. (J–K) Correlation analysis between gut microbiota and weight, RBG (J), and behavioral function (K), in impaired mice. (L) COG function classification. (n ​= ​8/group). Abbreviations: NC: normal control group; SE: normal control with semaglutide group; DM: Diabetes mellitus group; DM_SE: Diabetes mellitus with semaglutide group. Data are presented as mean ​± ​SD. ∗*p* ​< ​0.05, ∗∗*p* ​< ​0.01, ∗∗∗*p* ​< ​0.001, ∗∗∗∗*p* ​< ​0.0001.

Next, we investigated whether semaglutide altered the composition of the gut microbiota. Fecal samples were collected, and the composition of the gut microbiota was analyzed by 16S rRNA pyrosequencing. The obtained sequences had a mean length of 415 base pairs and were classified as 4518 ASVs. A total of 178 ASVs were shared among all samples, while over 84 ​% of the ASVs were unique to the different groups of mice. This indicates that only 3.9 ​% of the observed ASVs were unaffected by drug treatment or diabetes modeling, suggesting significant differences among the four groups (Fig. 4A). The α-diversity and β-diversity analysis revealed significant differences in microbial abundance among the groups. The α-diversity and β-diversity of DM group mice were significantly reduced (*p* ​< ​0.001, *p* ​< ​0.0001) (Fig. 4B and C), while the β-diversity of DM-SE group mice increased compared to that of DM group mice (*p* ​< ​0.05) (Fig. 4C), indicating that SE treatment can improve the gut microbiota composition in DM mice. The PcoA analysis further confirmed significant alterations in gut microbiota composition across the different groups (*R* ​= ​0.9742, *p* ​= ​0.001) (Fig. 4D). Fig. 4E shows the microbial composition at the family level, while Fig. 4F shows the dominant species at the genus level in each group of mice. LEfSe and linear discriminant analysis (LDA) (Fig. 4H) were used to identify specific taxa (from domain to genus level) that were significantly altered among the groups.

At the genus level, the relative abundances of *g_Alistipes*, *g_norank_f_Eubacterium_coprostanoligenes_group,* and *g_Parabacteroides* significantly decreased in DM mice, but semaglutide treatment reversed this trend. Semaglutide also significantly decreased the relative abundances of *g_faecalibaculum*, *g_Colodertribacter*, *g_GCA-900066575*, *g_Erysipelatoclostridium*, *g_norank_f_Lachnospiraceae* in DM mice (Fig. 4I). These results suggest that semaglutide intervention can significantly restore gut microbiota disruption caused by DM.

Furthermore, the identified semaglutide-related genera were correlated with cognition, obesity-associated weight, and random blood glucose (RBG) levels using Spearman analyses (Fig. 4J and K). As shown in Fig. 4J, *Prevotellaceae_NK3B31_group*, *g_norank_f_Eubacterium_coprostanoligenes_group*, *Clostridium_sensu_strito_1*, *Ruminococcus*, *Coriobacteriaceae_UGC-002*, and *Eubacterium_xyanophilum_group* were associated with weight, whereas *norank_f_Muribaculaceae*, *Prevotellaceae_NK3B31_group*, *Faecalibaculum*, *Prevotellaeae_UCG-001*, *Colidextribacter*, *Blautia*, *Coriobacteriaceae_UGC-002*, and *Eubacterium_xyanophilum_group* were correlated with RBG. In addition, *norank_f_Muribaculaceae*, *Prevotellaceae_NK3B31_group*, *Prevotellaeae_UCG-001*, *g_norank_f_Eubacterium_coprostanoligenes_group*, *Ruminococcus* positively correlated with cognitive ability, while *Faecalibaculum*, *Colidextribacter*, *Coriobacteriaceae_UGC-002*, *Akkermansia*, *GCA-900066575* were negatively associated with cognitive abilities (Fig. 4K). Thus, the beneficial effects of semaglutide on cognitive function may be associated with the maintenance of gut microbiota homeostasis.

As shown in Fig. 4L, COG functional classification analysis revealed the presence of gut bacteria in the four groups of fecal samples. The biological processes involved in the gut microbiota of the DM_SE group were more similar to those of the NC group than to those of the DM group.

### The effects of semaglutide treatment on fecal metabolic profiles

**Fig. 5:**
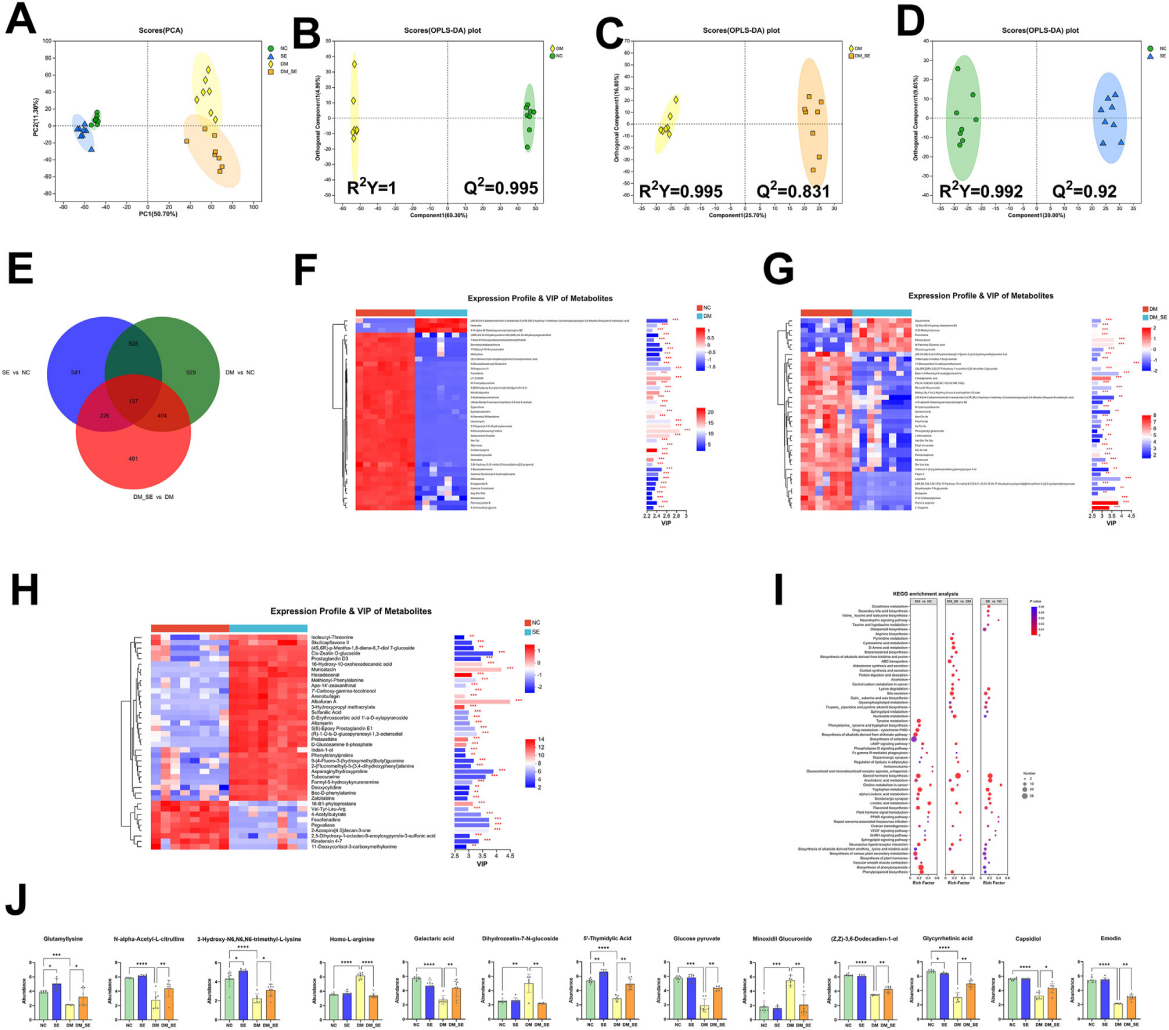
Semaglutide treatment regulated differential microbial metabolites in feces. (A) PCA analysis of feces. OPLS-DA of feces between NC and DM groups (B), DM and DM_SE groups (C). NC and SE groups (D). (E) Venn analysis. VIP scores analysis of NC and DM (F), DM and DM_SE (G), NC and SE groups (H). (I) Selected potential metabolites biomarkers. (J) Bubble diagram of enriched KEGG pathways. (n ​= ​7–8/group). Abbreviations: NC: normal control group; SE: normal control with semaglutide group; DM: Diabetes mellitus group; DM_SE: Diabetes mellitus with semaglutide group. Data are presented as mean ​± ​SD. ∗*p* ​< ​0.05, ∗∗*p* ​< ​0.01, ∗∗∗*p* ​< ​0.001, ∗∗∗∗*p* ​< ​0.0001.

After observing the changes in the gut microbiota, we explored whether there were changes in fecal metabolites. The fecal metabolites in the four groups were analyzed using UHPLC-MS/MS, identifying a total of 4547 metabolites, including 2692 positive metabolites and 1855 negative metabolites. Of these, 1958 metabolites were annotated in the KEGG database (43 ​%). PCA was used to assess the overall metabolic differences between the groups of samples. As shown in Fig. 5A, the metabolites in the four groups were clearly distinguished, with the first principal component accounting for 50.7 ​% of the total metabolites. To better differentiate between regional groups and improve the effectiveness and parsing ability of the model, we used OPLS-DA. As shown in Fig. 5B–D, the comparisons between DM vs. NC, DM_SE vs. DM, and SE vs. NC revealed significant separation, with high *R*^*2*^*Y* and *Q*^*2*^ values verifying the reliability of the model. Venn analysis was used to further identify differential metabolites, applying the screening criteria of *p-*value <0.05, VIP scores >1, and FC ​> ​1. The number of differential metabolites in the comparisons of DM vs. NC, DM_SE vs. DM, and SE vs. NC was 1998, 1258, and 1432, respectively (Fig. 5E). VIP score analysis further identified differential metabolites, focusing on those with VIP values greater than 2. As shown in Fig. 5F–H, the top 40 differential metabolites are listed. In the NC and DM groups, typical metabolites include *N*-Docosahexaenoyl Glutamine (VIP ​= ​2.45), Gamma-Glutaminyl-4-hydroxybenzene (VIP ​= ​2.41), Ser-Tyr (VIP ​= ​2.40). In the DM_SE group compared to DM, key differential metabolites include Glucose pyruvate (VIP ​= ​3.03), Minoxidil Glucuronide (VIP ​= ​3.47), Dihydrozeatin-7-*N*-glucoside (VIP ​= ​3.66), Homo-L-arginine (VIP ​= ​3.82). Between SE and NC, differential metabolites include 16-Hydroxy-10-oxohexadecanoic acid (VIP ​= ​3.54), D-Glucosamine 6-phosphate (VIP ​= ​2.80) and Asparaginylhydroxyproline (VIP ​= ​3.87).

According to OPLS-DA and VIP scores analysis, potential biomarkers including Glutamyllysine, *N*-alpha-Acetyl-L-citrulline, 3-Hydroxy-N6,N6,N6-trimethyl-L-lysine, Galactaric acid, 5′-Thymidylic acid, Glucose pyruvate, (Z,Z)-3,6-Dodecadien-1-ol, Glycyrrhetinic acid, Capsidiol, Emodin were significantly decreased in DM mice compared to NC (*p* ​< ​0.05). These trends were reversed by semaglutide treatment in DM_SE (Fig. 5J). The abundance of Homo-L-arginine, Dihydrozeatin-7-*N*-glucoside Minoxidil Glucuronide increased in the DM group, but decreased after semaglutide treatment (*p* ​< ​0.05).

Differential metabolites were subjected to enrichment analyses using the Kyoto Encyclopedia of Genes and Genomes database. As shown in Fig. 5I, common pathways co-enriched in the DM vs NC, DM_SE vs DM, and SE vs NC comparisons include Steroid hormone biosynthesis (map00140), Tryptophan metabolism (map00380), Linoleic acid metabolism (map00591), Sphingolipid signaling pathway (map 04071), Neuroactive ligand-receptor interaction (map 04080), Arachidonic acid metabolism(map 00590), and Phenylpropanoid biosynthesis (map 00940).

### The effects of semaglutide treatment on blood metabolic profiles

**Fig. 6:**
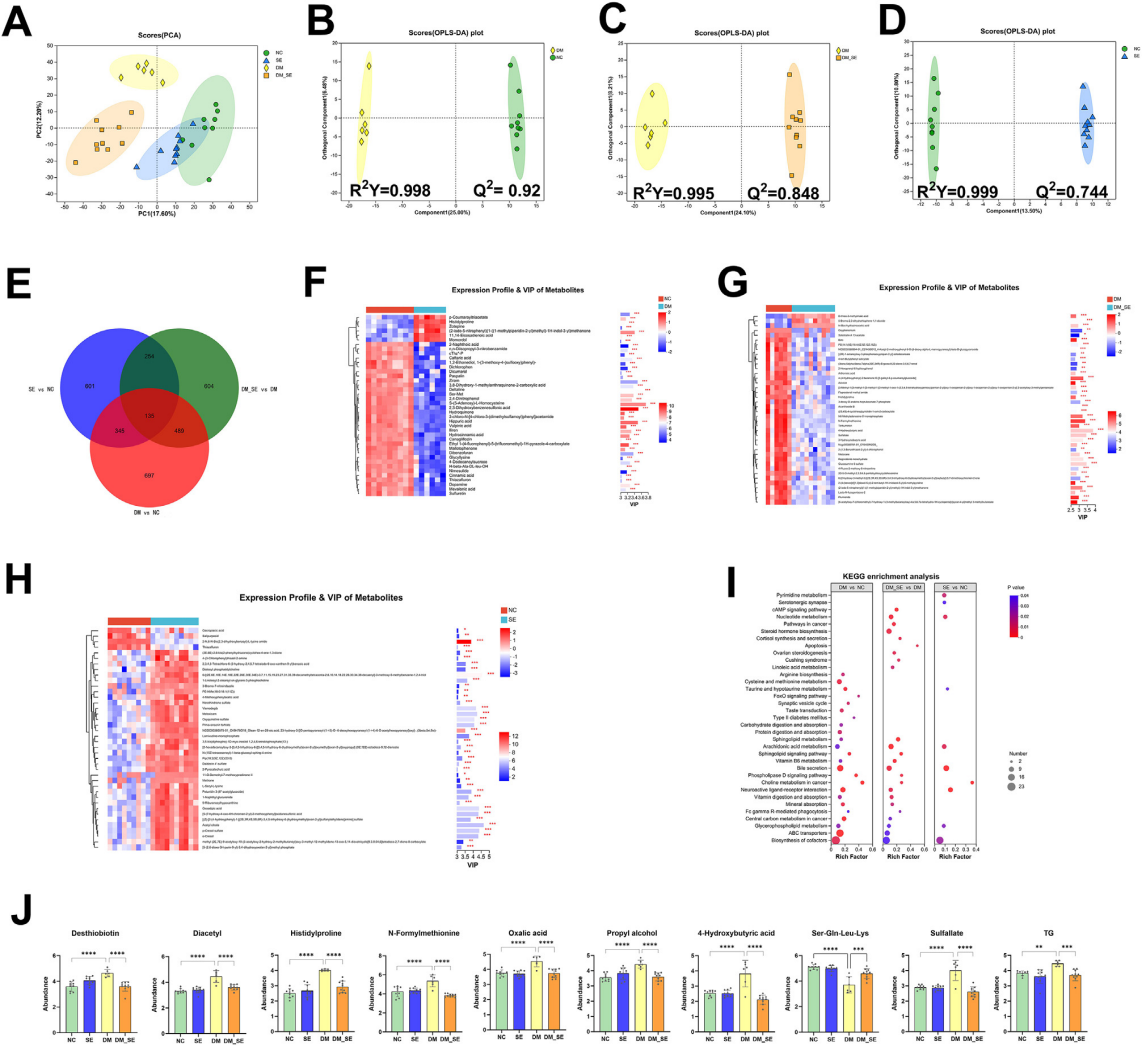
Semaglutide treatment regulated differential microbial metabolites in serum. (A) PCA analysis in serum. OPLS-DA in serum between NC and DM groups (B), DM and DM_SE groups (C), NC and SE groups (D). (E) Venn analysis. VIP score analysis of NC and DM (F), DM and DM_SE (G), NC and SE groups (H). (I) Selected potential metabolites biomarkers. (J) Bubble diagram of enriched KEGG pathways. (n ​= ​6–10/group). Abbreviations: NC: normal control group; SE: normal control with semaglutide group; DM: Diabetes mellitus group; DM_SE: Diabetes mellitus with semaglutide group. Data presented as mean ​± ​SD. ∗*p* ​< ​0.05, ∗∗*p* ​< ​0.01, ∗∗∗*p* ​< ​0.001, ∗∗∗∗*p* ​< ​0.0001.

Blood metabolic variations in the comparisons of DM vs. NC, DM_SE vs. DM, and SE vs. NC were obtained using UHPLC-MS/MS. A total of 3116 metabolites were identified. PCA was performed to gain a preliminary understanding of the overall metabolic differences among each group and the degree of variation among samples within each group. PCA analysis showed significant differences among all groups (Fig. 6A). OPLS-DA was then used to further explore potential biomarkers following semaglutide treatment. The corresponding *R*^*2*^*Y* values were 0.998, 0.995, and 0.999, respectively, and the corresponding *Q*^*2*^ values were 0.92, 0.848, and 0.774, respectively, in the comparisons of DM vs. NC, DM_SE vs. DM, and SE vs. NC, respectively, indicating the reliability of the proposed models (Fig. 6B–D). We used a *p*-value <0.05, VIP score >1, and FC ​> ​1 as the conditions for screening differential metabolites for the Venn analysis. As shown in Figs. 6E, 1666 differential metabolites were detected between the NC and DM groups. Relative to DM and NC, 1482 and 1335 differential metabolites were obtained in the DM_SE and DM_SE groups, respectively. Based on the above results, we conducted a cluster analysis using the top 40 differential metabolites with VIP scores greater than 2 (Fig. 6F–H). More than 100 differential metabolites had VIP scores >2.0 in the DM group relative to NC, in DM_SE mice relative to DM mice, and in SE mice relative to NC mice. The typical differential metabolites in VIP scores analysis in DM included histidylproline (VIP ​= ​3.15), 2,3-Dihydroxybenzenesulfonic acid (VIP ​= ​3.37), hydroquinone (VIP ​= ​3.51), and dopamine (VIP ​= ​3.03) when compared to NC. The typical differential metabolites in VIP scores analysis in DM_SE included 4-Hydroxybutyric acid (VIP ​= ​3.86), N -Formylmethionine (VIP ​= ​3.37), Sulfallate (VIP ​= ​3.44), histidylproline (VIP ​= ​2.79) when compared to DM. The typical differential metabolites in VIP scores analysis in SE included Oxoadipic acid (VIP ​= ​4.36), Acetyl citrate (VIP ​= ​4.65), and p-Cresol sulfate (VIP ​= ​4.38) when compared to NC.

According to OPLS-DA and VIP score analysis, potential biomarkers, such as desthiobiotin, diacetyl, histidylproline, *N*-Formylmethionine, oxalic acid, propyl alcohol, 4-Hydroxybutyric acid, sulfate, and TG, were significantly increased in DM mice relative to NC, and these trends were reversed by semaglutide treatment in DM_SE (Fig. 6J). The abundance of ser-gln-leu-lys decreased in the DM group, and semaglutide treatment increased their abundance (*p* ​< ​0.001).

Similarly, the differential metabolites between the groups were used for enrichment analysis in the KEGG database. As shown in Fig. 6I, the differential metabolites between the NC and DM groups were mainly enriched in ABC transporters (map 02010), bile secretion (map 04976), neuroactive ligand-receptor interactions (map 04080), and central carbon metabolism in cancer (map 05230). For the comparison of DM_SE and DM, pathways mainly involved in sphingolipid metabolism (map 00600), the sphingolipid signaling pathway (map 04071), the cAMP signaling pathway (map 04024), and neuroactive ligand-receptor interaction (map 04080). In the comparison of SE and NC, regulated metabolites were mainly enriched in choline metabolism in cancer (map 05231), bile secretion (map 04976), and neuroactive ligand-receptor interaction (map 04080). These differential metabolites in the blood were co-enriched in the same pathways, such as bile secretion, neuroactive ligand-receptor interaction, and biosynthesis of cofactors.

### The effects of semaglutide treatment on the cerebral transcriptome

**Fig. 7:**
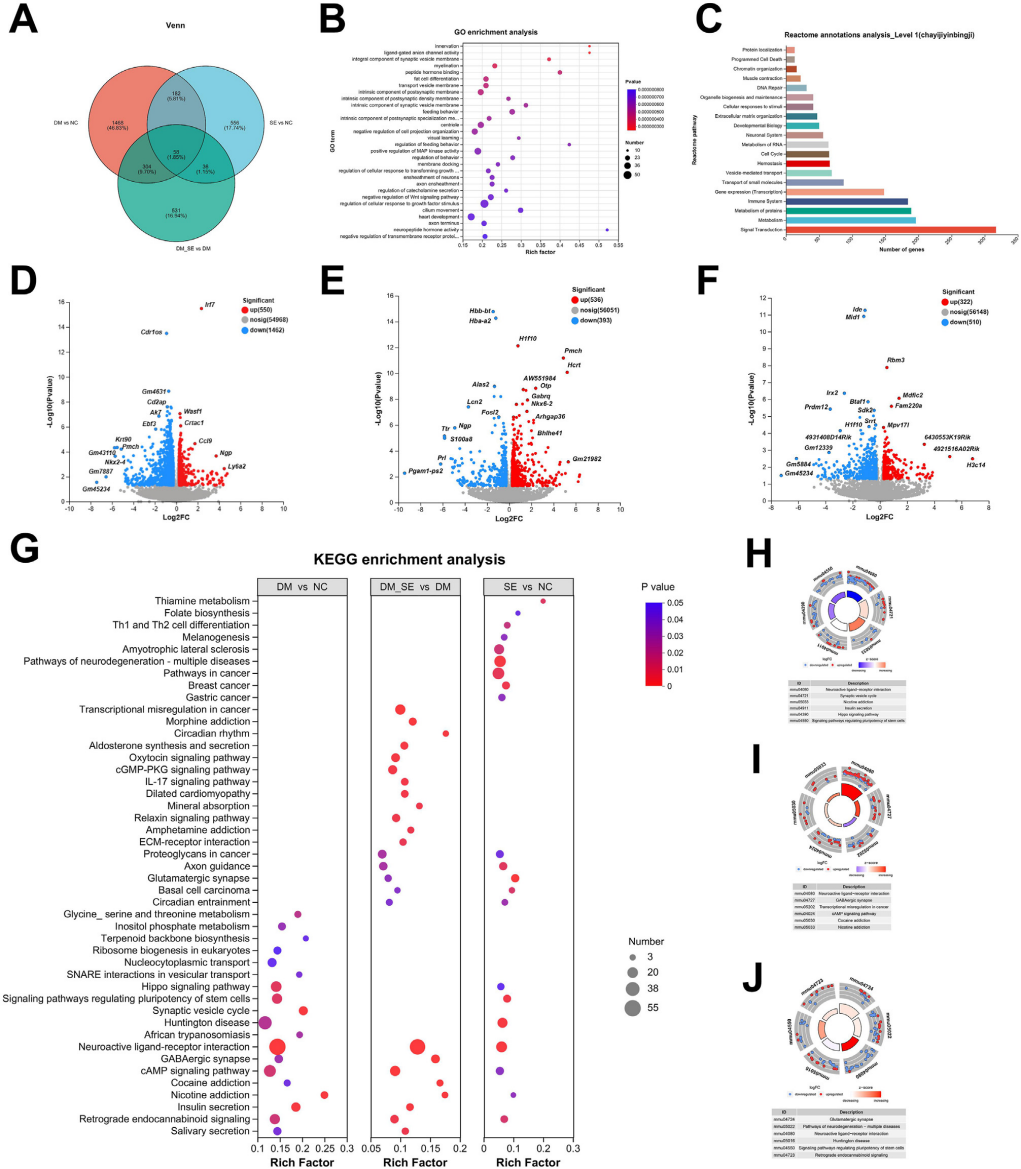
Effect of semaglutide on brain transcriptome. (A) Venn analysis. (B) GO enrichment analysis of DEGs. (C) Reactome annotations analysis. (D–F) Volcano plots of significant DEGs for DM vs. NC (D), DM_SE vs. DM (E), and SE vs. NC (F). Red dots indicate upregulated genes; blue dots indicate downregulated genes. (G) Bubble diagram of KEGG enrichment analysis. (H–I) Circle diagram of KEGG enrichment analysis for DM vs. NC (H), DM_SE vs. DM (I), and SE vs. NC (J). (n ​= ​4/group). Abbreviations: NC: normal control group; SE: normal control with semaglutide group; DM: Diabetes mellitus group; DM_SE: Diabetes mellitus with semaglutide group.

RNA-Seq analysis was performed on cerebral samples from the NC, SE, DM, and DM_SE groups. A total of 46.35–75.55 million clean reads were detected for each sample after quality control. Venn analysis identified 2012 DEGs in the DM group compared to the NC group (Fig. 7A), with 550 genes upregulated and 1462 downregulated (Fig. 7D). Relative to the DM group, 929 DEGs were detected in the DM_SE group, including 536 upregulated and 393 downregulated genes (Fig. 7E). Semaglutide also induced 832 DEGs in the SE group relative to those in the NC group (Fig. 7F). GO enrichment analysis revealed that the significant DEGs that were primarily enriched in processes related to innervation, ligand-gate anion channel activity, integral components of synaptic vesicle membranes, myelination, transport vesicle membranes, intrinsic components of postsynaptic density membranes, and visual learning (Fig. 7B). Reactome annotation analysis of the DEGs revealed that they were mainly enriched in signal transduction, metabolism, gene expression (transcription), and vesicle-mediated transport (Fig. 7C).

Enrichment analysis of the DEGs between each group was conducted using the KEGG database (Fig. 7G). The most abundant DEGs were identified in the DM vs. NC comparison, with neuroactive ligand-receptor interactions, the synaptic vesicle cycle, nicotine addiction, and insulin secretion as the main biological processes (Fig. 7H). In the DM_SE vs. DM comparison, enriched pathways included neuroactive ligand-receptor interactions, GABAergic synapses, transcriptional misregulation in cancer, and the cAMP signaling pathway (Fig. 7I). In the SE group compared to the NC group, the DEGs were mainly enriched in glutamatergic synapses, pathways of neurodegeneration associated with multiple diseases, and neuroactive ligand-receptor interactions (Fig. 7J). Notably, neuroactive ligand-receptor interaction processes were enriched in all three comparisons, indicating that semaglutide treatment significantly influenced these interactions in both DM and NC mice. In the neuroactive ligand-receptor interaction pathway, the DEGs in the DM group were mostly downregulated compared to those in the NC group, whereas the DEGs in the DM_SE group were mostly upregulated compared to those in the DM group. This suggests that semaglutide improves cognitive function in DM mice by reversing the dysregulation of the neuroactive ligand-receptor interaction pathway.

### Correlation analysis

**Fig. 8:**
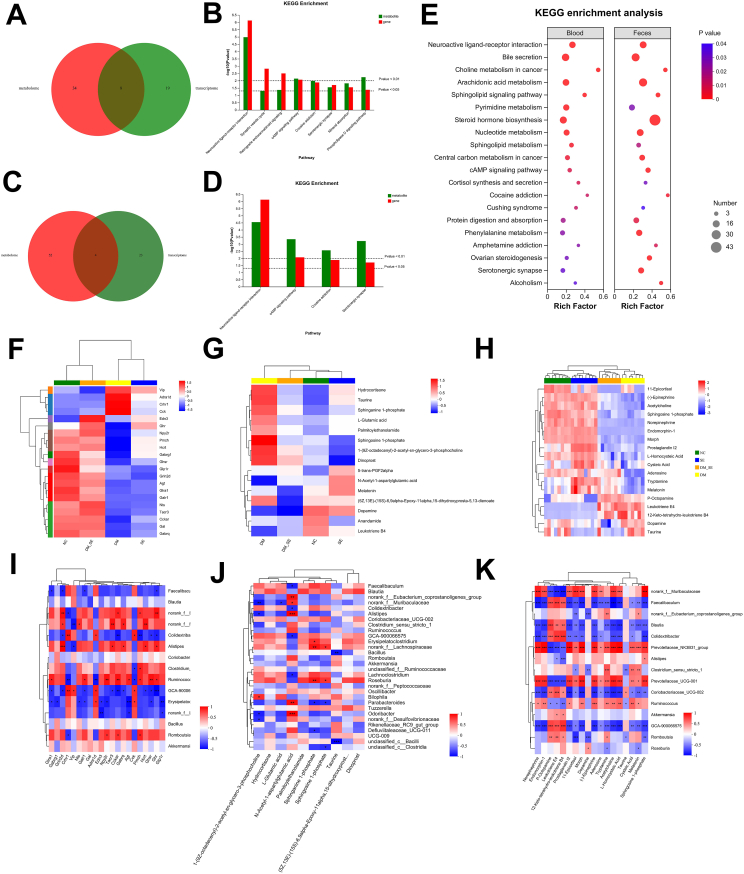
Integration of correlation analysis for metabolites, microbes and genes. (A) Venn diagram of common significant KEGG enrichment pathways for blood metabolic profiles and DEGs. (B) Specific common significant KEGG enrichment pathways for blood metabolic profiles and DEGs. (C) Venn diagram of common significant KEGG enrichment pathways for feces metabolic profiles and DEGs. (D) Specific common significant KEGG enrichment pathways for blood metabolic profiles and DEGs. (E) Bubble diagram of KEGG enrichment analysis for blood and fecal metabolic profiles. (F) Heat map of DEGs involved in neuroactive ligand-receptor interaction. (G) Heat map of differential serum metabolites involved in neuroactive ligand-receptor interaction. (H) Heat map of differential fecal metabolites involved in neuroactive ligand-receptor interaction. (I) Spearman correlation analyses of DEGs and differential serum metabolites involved in neuroactive ligand-receptor interaction. (J) Spearman correlation analyses of DEGs and differential fecal metabolites involved in neuroactive ligand-receptor interaction. (K) Spearman correlation analysis of gut microbiota and DEGs involved in neuroactive ligand-receptor interaction. (L) Spearman correlation analysis of gut microbiota and differential serum metabolites involved in neuroactive ligand-receptor interaction. (M) Spearman correlation analysis of gut microbiota and differential fecal metabolites involved in neuroactive ligand-receptor interaction. Red indicates a positive correlation; blue indicates a negative correlation. Abbreviations: NC: normal control group; SE: normal control with semaglutide group; DM: Diabetes mellitus group; DM_SE: Diabetes mellitus with semaglutide group. ∗*p* ​< ​0.05, ∗∗*p* ​< ​0.01, ∗∗∗*p* ​< ​0.001, ∗∗∗∗*p* ​< ​0.0001.

In-depth analyses were conducted by integrating DEGs (|log2FC|≥1.2 and *p* ​< ​0.05) and differential metabolites (VIP ≥1 and *p* ​< ​0.05) from blood and fecal metabolic profiles. As shown in Fig. 8A, eight common KEGG enrichment pathways (*p* ​< ​0.05) were identified between the blood metabolic profiles and DEGs in the cerebral transcriptome. Fig. 8B highlights these common pathways, such as the neuroactive ligand-receptor, synaptic vesicle cycle, and cAMP signaling pathways. In addition, four common KEGG pathways (*p* ​< ​0.05) were identified between the fecal metabolic profiles and DEGs in the cerebral transcriptome (Fig. 8C), including the neuroactive ligand-receptor, cAMP signaling pathway, cocaine addiction, and serotonergic synapses (Fig. 8D). Fig. 8E shows the common pathways between the blood and fecal metabolic profiles.

Furthermore, considering the blood metabolome, fecal metabolome, and transcriptome, semaglutide is speculated to alleviate cognitive impairment in DM mice by modulating the neuroactive ligand-receptor pathway. To further verify this observation, the key DEGs and metabolites (blood and feces) enriched in the neuroactive ligand-receptor pathway were analyzed and presented using heat maps (Fig. 8F–H). As shown in Fig. 8F–H, the clustering of the four groups differed significantly. To further investigate the role of the gut microbiota in cognitive function, we conducted a correlation analysis between DEGs or differential metabolites in the neuroactive ligand-receptor pathway and the gut microbiota. As shown in Figs. 8I, 73 significant DEGs–microbiota correlation pairs were detected, with 35 pairs being significantly positively correlated and 38 pairs being negatively correlated. In the analysis of blood metabolites and microbiota, 26 correlation pairs were identified, with 11 positive and 15 negative correlations (Fig. 8J). In total, 160 significant correlation pairs were detected in the analysis of fecal metabolites and microbiota (Fig. 8K). These results indicate that the gut microbiota is closely related to the genes and metabolites enriched in the neuroactive ligand-receptor pathway.

### Integrated multi-OMICs analysis on semaglutide treatment

**Fig. 9:**
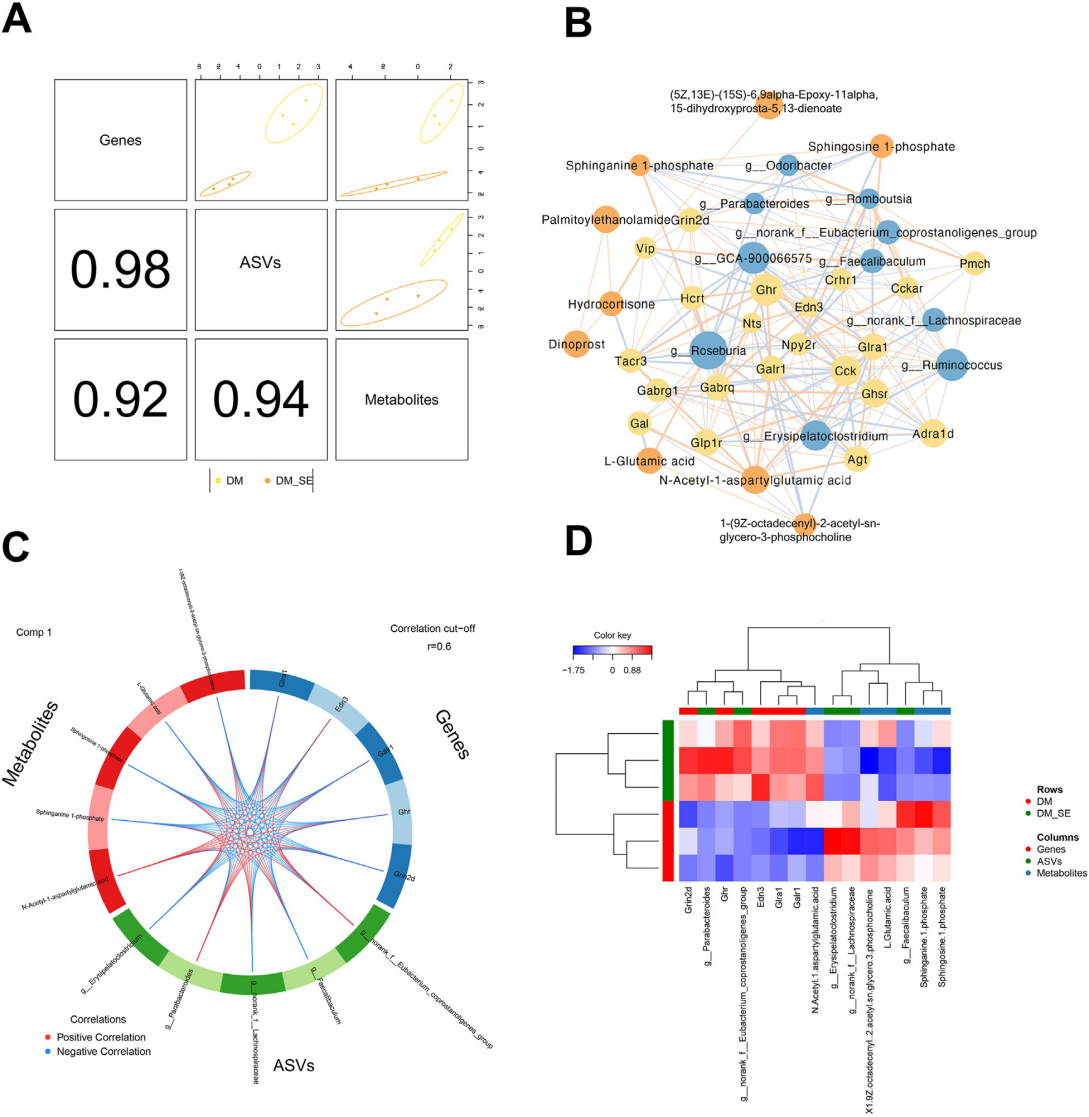
Integrated multi-OMICs analysis of semaglutide treatment. (A) Diagnostic plot Samples were colored by grouping. The ellipse represents a 95 ​% confidence level ellipse plot. The number in the bottom left corner represents the correlation coefficient between the first principal component of each dataset. (B) Correlation Network Analysis showed correlations between selected signatures. The yellow, blue, and orange dots represent genes, metabolites, and microbes, respectively. The node size represents the magnitude of the central coefficient value, which is positively correlated. The thickness of the line indicates the magnitude of the correlation coefficient, with yellow indicating positive correlation and blue indicating negative correlation (C) The Circos plot shows positive (negative) correlations with a correlation cut-off (r ​= ​0.6), denoted by red (blue) lines, between selected signatures. (D) The clustered heatmap of selected signatures from each omics dataset. Rows represent samples, and columns represent features. Abbreviations: NC: normal control group; SE: normal control with semaglutide group; DM: Diabetes mellitus group; DM_SE: Diabetes mellitus with semaglutide group.

After identifying the different omics signatures that were upregulated or downregulated in key signaling pathways of semaglutide-treated DM mice, we evaluated the interaction between gene expression in the cortex (Fig. 7), gut microbiota (Fig. 4), and microbial metabolites (Fig. 5, Fig. 6), in relation to the impact of semaglutide treatment on cognitive impairment in DM mice (Fig. 1). By integrating the gene-microbiota and metabolite-microbiota correlation pairs, candidate genes (n ​= ​21), metabolites (n ​= ​10), and genera (n ​= ​10) were identified as key predictors from each omics dataset. The latent component method (DIABLO) was used for multi-OMIC data integration analysis to identify biomarkers. The predictors were highly correlated and contributed to the significant separation between the DM and DM_SE groups (Fig. 9A). Network correlation analysis revealed a close correlation between different types of signatures (Fig. 9B). A correlation greater than 0.6 between different types of signatures is shown in Fig. 9C. Based on betweenness centrality, the expression or abundance of N_Acetyl_1_ aspartylglutamic acid, *Galr1*, *Glra1,* and *g_Erysipelatoclostridium* may play key roles in the network. Compared to DM mice, the *Grin2d*, *g_parabacteroides*, *Ghr*, *g_norank_f_Eubacterium_coprostanoligenes_group*, *Edn3*, *Glra1*, *Galr1*, N_Acetyl_1_aspartylgutamic_acid were significantly increased, while the expression or abundance of g_Erysipelatoclostridium, g_norank_f_Lachnospiraceae, X1_9Z_octadecenyl_2_acetyl_sn_glycero_3_phosphocholine, L_Glutamic_acid, g_Faecalibaculum, Sphinganine_1_phosphate and Sphingosine_1_phosphate were significantly decreased (Fig. 9D).

## Discussion

In this study, we provided evidence demonstrating the beneficial effects of semaglutide treatment in alleviating cognitive dysfunction. Semaglutide treatment ameliorated gut dysbiosis in STZ-induced diabetic mice. Furthermore, we observed that semaglutide significantly altered the neuroactive ligand-receptor interaction pathway in plasma metabolic profiles, fecal metabolic profiles, and the cerebral transcriptome. These findings suggest the potential of semaglutide therapy as a probiotic intervention for diabetes-related cognitive impairment.

Semaglutide (C_187_H_291_N_45_O_59_) is a long-acting GLP-1 receptor agonist with 94 ​% sequence homology to native GLP-1 [28]. Compared to native GLP-1, semaglutide has a similar ability to activate the GLP-1 receptor, but its half-life is longer due to its resistance to dipeptidyl peptidase IV [29]. Recently, the effects of semaglutide on the central nervous system have received widespread attention, and it has become increasingly clear that semaglutide exerts neuroprotective effects. However, the underlying molecular mechanism remains unclear. Previous studies have shown that semaglutide ameliorates cognitive and glucose metabolism dysfunction in AD mice [30], reduces inflammation and apoptosis, and stabilizes neurogenesis in a stroke model [31]. GLP-1 receptors are present in the brain, including the cerebral cortex and hippocampus, and semaglutide can interact with these receptors [[32], [33], [34]]. Therefore, we explored the neuroprotective effects of semaglutide and investigated the metabolomic, transcriptomic, and microbiological mechanisms involved using a mouse model of diabetes. In the MWM tests, semaglutide reversed STZ-induced impairment of spatial learning and memory. Furthermore, subsequent evaluation revealed that the subcutaneous injection of semaglutide significantly hindered the loss of hippocampal neurons in the CA1 and CA3 regions of DM mice, which are key factors in hippocampal-dependent cognitive function. Similarly, TEM revealed that damage to synapses, including an increase in synaptic width and a decrease in PSD thickness, was alleviated by semaglutide. Synaptic plasticity is widely believed to be crucial for biological processes in learning and memory. Consistent with our results, STZ-induced diabetic mice have cognitive impairment [35,36]. In addition, semaglutide increases the number of hippocampal neurons in the CA1 and CA3 subregions in a rat stroke model [31]. Furthermore, DM mice show markedly activated astrocytes and microglia in the hippocampus and cortex. Interestingly, this effect was significantly inhibited by semaglutide treatment. These observations suggest that semaglutide substantially ameliorates cognitive impairment in mice with DM.

The gut microbiota regulates the host brain function and cognitive behavior. Disruptions in gut microbes also contribute to the pathogenesis of neurodegenerative diseases [37]. The results of this study indicate that DACD mice exhibit a significant gut microbial disorder. Interestingly, this effect was partially reversed with semaglutide treatment. We found that semaglutide treatment prevented a significant decrease in the abundance of *g_Alistipes* in DM mice. *Alistipes* play an important role in maintaining intestinal integrity and cognitive function [38]. The abundance of *Alistipes* decreases significantly at the order, family, and genus levels in cognitively impaired mice [39]. A previous study showed that leucine restriction (LR) improved HFD-induced cognitive impairment and increased the abundance of *Alistipes,* which can produce short-chain fatty acid (SCFA) [40]. In addition, Dendrobium officinale polysaccharide treatment was shown to alleviate AD-related cognitive decline and reshape the perturbation of gut microbe, including upregulating the abundance of *Alistipes* [41]. Furthermore, Stefano et al. reported that insulin degludec/liraglutide (another GLP-1 analog) alters the gut microbiome of older patients with type 2 diabetes, and an increased abundance of *Alistipes* was directly associated with cognitive function [42].

Moreover, LEfSe analysis revealed that semaglutide treatment increased the relative abundance of *g_norank_f_Eubacterium_coprostanoligenes, g_Bacteroides, and g_Parabacteroides*. Supplementation with Mexican functional foods has been reported to improve cognitive behavior and increase the abundance of *g_norank_f_Eubacterium_coprostanoligenes* which are involved in multiple beneficial metabolic effects [43]. *Eubacterium_coprostanoligenes* may also enhance the integrity of the intestinal mucus barrier by promoting mucin secretion [44] and mediate dyslipidemia through sphingosine [45]. In our study, we found a positive correlation between *g_norank_f_Eubacterium_coprostanoligenes* and cognitive function. *Bacteroides*, a SCFA-producing bacterium, has potent immunomodulatory and anti-inflammatory effects [46]. A previous study showed that *Bacteroides* is decreased in patients with AD [47]. It has also been reported that *Bacteroides* was related to infant composite cognition [48] and is positively associated with subsequent neurodevelopment in male infants [49]. A recent study showed that *Parabacteroides* is positively correlated with cognitive function [50]. *Parabacteroides* can regulate propanoic, citramalic, and acetic acids, significantly improving cognitive abilities [51]*.* Interestingly, fecal *Alistipes*, *Parabacteroides*, and *Bacteroides* were significantly enriched in centenarians compared to younger controls, indicating that these may be potential markers of successful aging [52].

Additionally, we found that *g__Faecalibaculum*, *g__unclassified_f__Lachnospiraceae*, *g__Coriobacteriaceae_UCG-002*, *g__Colidextribacter*, *g__GCA-900066575*, *g__Erysipelatoclostridium*, *g__norank_f__Lachnospiraceae* were significantly increased in DM mice, and semaglutide treatment partly reversed these changes. This suggests that semaglutide helps maintain a stable gut microbiota. A high-sugar diet can promote the growth of *g_Faecalibaculum*, which can replace normal gut bacteria and cause an imbalance in the gut microbiota [53]. It has been shown that *Lachnospiraceae* is associated with the odds of amyloid positivity and p-tau status [54]. Previous studies have demonstrated that *g__Coriobacteriaceae_UCG-002* is a harmful bacterium correlated with abnormal metabolism and inflammation [55,56]. Additionally, *g__Colidextribacter* has been reported to be positively associated with pro-inflammatory factors and negatively correlated with the activities of antioxidant enzymes, with a significant increase observed in HFD-treated mice [57,58]. Moreover, *Erysipelatoclostridium* has also been identified as a harmful bacterium [35], and as a hub gene of AD, it is associated with AD via interactions mediated by fecal deoxycholic acid [59].

Furthermore, correlation and multi-OMIC analyses revealed strong links between microbial metabolites and semaglutide-induced alterations in the microbiome profile, and cortex neuroactive ligand receptor-related gene expression. These findings suggest a correlation between the beneficial effect of semaglutide on DACD and the gut-metabolism-brain axis. In this study, we observed that semaglutide altered the expression of neuroactive ligand receptor-related genes, including *Grin2d, Ghr, End3, Glra1, and Galr1*. The *Grin2d* gene, which encodes the GluN2D subunit of the glutamate receptor *N*-methyl-D-aspartate (NMDA), plays a central role in learning, memory, neuronal development, and postsynaptic signaling [60,61]. In a genome-wide association study of Alzheimer's disease, it was found that the expression of the brain messenger glutamate receptor *Grin2d* was reduced, potentially leading to impaired synaptic function, which is crucial for communication between nerve cells in the brain [62]. *Ghr* (growth gene) encodes a transmembrane GH receptor that modulates synaptic function, glutamatergic neurotransmission, and neural plasticity in the hippocampus, promoting cognition and memory [[63], [64], [65]]. *End3* encodes a member of the (ET) protein family. Endothelin is an endothelial-derived vasoactive peptide involved in various biological processes. Research has shown that ET-3 has anti-inflammatory effects, such as attenuating platelet-activating factor (PAF)-induced inflammation [66,67]. In our study, we found that semaglutide increased the expression of *End3*, suggesting that semaglutide has an anti-inflammatory effect associated with PAF. *Glra1* encodes a subunit of the inhibitory glycine receptor, mediating fast synaptic inhibition in the central nervous system [68]. Galr1 (Galanin receptor 1) is encoded by the *Galr1* gene. Upon binding to Galr1, galanin regulates insulin secretion [69], promotes growth hormone release [70], and affects cognitive performance [71]. In our study, we found that semaglutide promoted the expression of *Galr1*, indicating that the regulatory effects of semaglutide on cognitive function may be multifaceted.

Metabolomic analysis showed that semaglutide treatment increased the level of *N*-acetyl aspartyl glutamic acid (NAAG) and decreased the levels of L-glutamate, 2-acetyl-1-(9Z-octadecenyl)-sn-glycero-3-phosphocholine, sphingosine-1-phosphate (S1P), and sphinganine-1-phosphate (SA1P). NAAG primarily functions by activating presynaptic metabotropic glutamate (mGlu) receptors, inhibiting the release of neurotransmitters, including glutamate, and reducing the excitatory neurotoxicity of glutamate [72]. In AD, NAAG levels in the most severely affected brain regions are significantly lower than those in healthy individuals [73]. Interestingly, some studies suggest that increasing endogenous NAAG or activating the NAAG ligand mGlu3 is a promising strategy for treating neurodegenerative diseases [74,75]. Furthermore, semaglutide decreased glutamate levels. Glutamate is the main excitatory neurotransmitter in the central nervous system [76,77]. However, when synaptic glutamate concentrations increase excessively, they can induce excitotoxic cell death through NMDA receptor-mediated increases in intracellular Ca2^+^concentrations. Studies have shown that increased glutamate release within synaptic gaps and subsequent activation of glutamate receptors collectively mediate amyloid-beta protein-induced neurotoxicity [78]. In our study, we found that semaglutide intervention partially reversed the increase in glutamate and decrease in NAAG content in DM mice, suggesting that the glutamate circulatory system may play an important role in the cognitive improvement of semaglutide. 2-acetyl-1-(9Z-octadecenyl)-sn-glycero-3-phosphocholine is an intermediate in ether lipid metabolism, also known as PAF. PAF induces neurotoxicity [79] and may be involved in dementia [80]. Neurotoxic concentrations of glutamate can increase PAF content in the primary neuronal culture medium of the rat cerebral cortex [81]. In addition, PAF is an important pro-inflammatory mediator, which may be one mechanism through which it mediates neurotoxicity [82]. When inflammation intensifies, elevated PAF levels promote glutamate excitotoxicity and induce neurodegenerative changes [81]. These findings suggest that PAF amplifies the neuroinflammatory cascade associated with neurodegeneration and may be a potential biomarker of inflammation and neurodegeneration [83]. Sphingosine-1-phosphate (S1P) and Sphinganine-1-phosphate (SA1P) are both phosphate sphingolipids. The homeostasis of neural sphingolipids plays an important role in preventing synaptic loss, cell death, and neurodegenerative diseases [84]. Research has found that S1P can induce mitochondrial dysfunction, activate various inflammasomes, and increase the IL-1β formation [85]. Interestingly, research suggests that there are metabolic disorders of SA1P and S1P in both the brain and plasma in AD and confirms that SA1P and S1P in plasma and brain have great potential as biomarkers for AD [86,87]. In the present study, we found that semaglutide intervention improved sphingolipid metabolism disorders in DM mice, which may be one of the mechanisms through which GLP-1 improves cognitive impairment in DM mice.

In this study, we observed that semaglutide can maintain the homeostasis of gut microbiota and improve the cognitive ability of DM mice. Nevertheless, we cannot conclude that the gut microbiome plays a causative role in cognitive function. Moreover, we cannot conclude that the gut microbiome is the only factor mediating semaglutide improvement of cognitive abilities. A study showed that semaglutide can promote glucose metabolism through the SIRT1 pathway, thereby improving cognitive ability in AD mice [30]. Moreover, another study reported that semaglutide can improve autophagy flux, reduce oxidative stress, alleviate mitochondrial dysfunction, reduce inflammation, promote cellular neurogenesis, and activate dopamine synaptic pathways [88]. Therefore, it is worth noting that the improvement of cognitive function by semaglutide may involve multiple factors; however, in DM mice with cognitive impairment induced by HFD combined with STZ, gut microbiota is intricately involved in semaglutide enhancing cognitive function.

In conclusion, we identified a novel and compelling link between gut microbiota and cognitive function in DM mice treated with semaglutide. Specifically, Semaglutide treatment modulated gut microbiota composition and structure, altered microbial metabolites, regulated the expression of genes related to neuroactive ligand receptors, alleviated astrocyte activation and neuroinflammation, protected the chemical synapse structure, and improved cognitive function in DM mice. These findings suggest that semaglutide or similar stable GLP-1 analogs may be promising therapies to prevent the degenerative processes observed in cognitive impairment diseases such as DACD.

## Author contributions

Liqin Qi and Huimin Kang:Conceptualization, Funding acquisition and wrote the original draft. Feihui Zeng and Menglan Zhan: Methodology and Software. Cuihua Huang: Data curation. Qintao Huang and Lijing Lin: Supervision. Guanlian He: Writing, review, and editing. Xiaoying Liu and Xiaohong Liu: Resources. Libin Liu: Project administration.

## Declaration of Competing Interest

The authors have declared no competing interests.
